# Effects of Clopidogrel, Prasugrel and Ticagrelor on Microvascular Function and Platelet Reactivity in Patients With Acute Coronary Syndrome Undergoing Coronary Artery Stenting. A Randomized, Blinded, Parallel Group Trial

**DOI:** 10.3389/fcvm.2021.780605

**Published:** 2021-12-13

**Authors:** Boris Schnorbus, Kerstin Jurk, Karl J. Lackner, Caroline Welk, Thomas Münzel, Tommaso Gori

**Affiliations:** ^1^Zentrum für Kardiologie, Kardiologie I, Universitätsmedizin Mainz, Johannes Gutenberg-University Mainz, Mainz, Germany; ^2^Center for Thrombosis and Hemostasis, Universitätsmedizin Mainz, Johannes Gutenberg- University Mainz, Mainz, Germany; ^3^Institute of Clinical Chemistry and Laboratory Medicine, Universitätsmedizin Mainz, Johannes Gutenberg- University Mainz, Mainz, Germany; ^4^Deutsches Zentrum für Herz Kreislauf-Forschung (DZHK), Standort Rhein-Main, Partnereinrichtung Mainz, Mainz, Germany

**Keywords:** microvascular (MV), antiplatelet, coronary, stent, randomized

## Abstract

**Aims:** In this pre-specified analysis of the “endothelium, stent and antiplatelet therapy” study, we investigate the impact of antiplatelet therapies on microvascular function in patients undergoing stenting for an acute coronary syndrome.

**Methods and Results:** Fifty-six patients [age: 63(55–67) years, males, 10 diabetics, 27 non-ST-elevation myocardial infarction] were randomized to receive clopidogrel, ticagrelor or prasugrel in form of oral loading 2 h before stenting followed by oral therapy. Investigators were blinded to the allocation. Laser-Doppler microvascular function and ADP-induced platelet aggregation capacity were measured at baseline, 2 h after oral antiplatelet loading, and 1 day, 1 week and 1 month after stenting during chronic therapy with the same antiplatelet agent. Platelet aggregation decreased in all groups 2 h after oral loading, with a significantly larger effect in the prasugrel group (*P* = 0.009). Similarly, prasugrel and ticagrelor loading was followed by an increase in microvascular reactive hyperemia (*P* = 0.007 and *P* = 0.042 compared to clopidogrel). This effect disappeared one day after coronary intervention, with a significant decrease in the prasugrel group (*P* = 0.026). Similarly, analysis of microvascular conductance showed a larger increase in the prasugrel group 2 h after loading (*P* = 0.022 among groups), and a decrease in all groups after stenting.

**Conclusions:** Oral loading with prasugrel (and less consistently ticagrelor) is associated with improved microvascular function and stronger platelet inhibition in acute coronary syndrome patients. The microvascular effect was however lost 1 day after stenting and during subsequent follow-up. Further studies are necessary to clarify the the long-term effects and potential benefits of P2Y12 inhibitors on microvascular damage.

**ClINICALTRIALS.gov N°:** NCT01700322

**EUDRACT-N°:** 2011-005305-73.

## Introduction

Platelet activation is associated with the release of mediators of inflammation and oxidative stress, which stimulate leukocyte chemotaxis, aggregation and endothelial dysfunction [reviewed in (1)], thus impairing micro- and macrovascular function. As a consequence, along with their antithrombotic benefits, clopidogrel and other P2Y_12_ inhibitors have been shown to have an impact on endothelial and vascular function in patients with and without coronary artery disease (2–5).

These processes assume a particular importance in the setting of coronary stenting, and even more in patients with acute coronary syndromes (ACS). In the Protecting Microcirculation During Coronary Angioplasty (PROMICRO)-2 Randomized Study (6), the microvascular impairment induced by coronary stenting was reduced following antiplatelet therapy with prasugrel. As well, a linear correlation between platelet inhibition and PCI-induced coronary microvascular impairment could be shown (7). Given its more potent antiplatelet effect, prasugrel was associated with significantly lower microvascular resistances (7), which might in turn result in better myocardial perfusion. In another paper, no difference was shown between prasugrel and ticagrelor in preventing coronary microvascular injury following a ST-elevation myocardial infarction (8).

The EST (endothelium, stent, and antiplatelet therapy) study was designed to investigate the impact of clopidogrel, prasugrel and ticagrelor on multiple parameters of vascular and platelet function in a randomized, blinded fashion in a group of patients undergoing stenting (percutaneous coronary intervention, PCI) in the setting of an ACS. The main results of the study show that prasugrel, as compared to ticagrelor and clopidogrel, prevents PCI-induced endothelial dysfunction of conduit arteries and vascular inflammation (9). Goal of this pre-specified substudy was to test the impact of the three drugs on microvascular function.

## Methods

### Objectives and Design of the Study

The protocol of the EST, a three-arms, parallel design, randomized, investigator-blinded trial, is published in (10). The main results of the EST study are published in (9); the current study was limited to the cohort treated following the original study protocol [see (10) for description]. Briefly, patients with unstable angina or non-ST-elevation myocardial infarction (NSTEMI) undergoing coronary intervention with a drug-eluting stent were randomized to receive oral loading with clopidogrel 600 mg, prasugrel 60 mg or ticagrelor 180 mg followed by therapy (respectively, 75 mg od, 10 mg od, and 90 mg bid) with the same drug. None of the patients had received P2Y12 inhibitors before. Study drugs were prepared and packaged in identical anonymous boxes by the hospital pharmacy. Participants underwent 6 visits. Vascular and platelet function were measured at screening, 2 h after the loading dose, and 1 day, 1 week, 1 month after stenting. Randomization was performed at the end of the screening visit. Catheterization was performed in all cases immediately after the 2 h visit.

**Figure 1:**
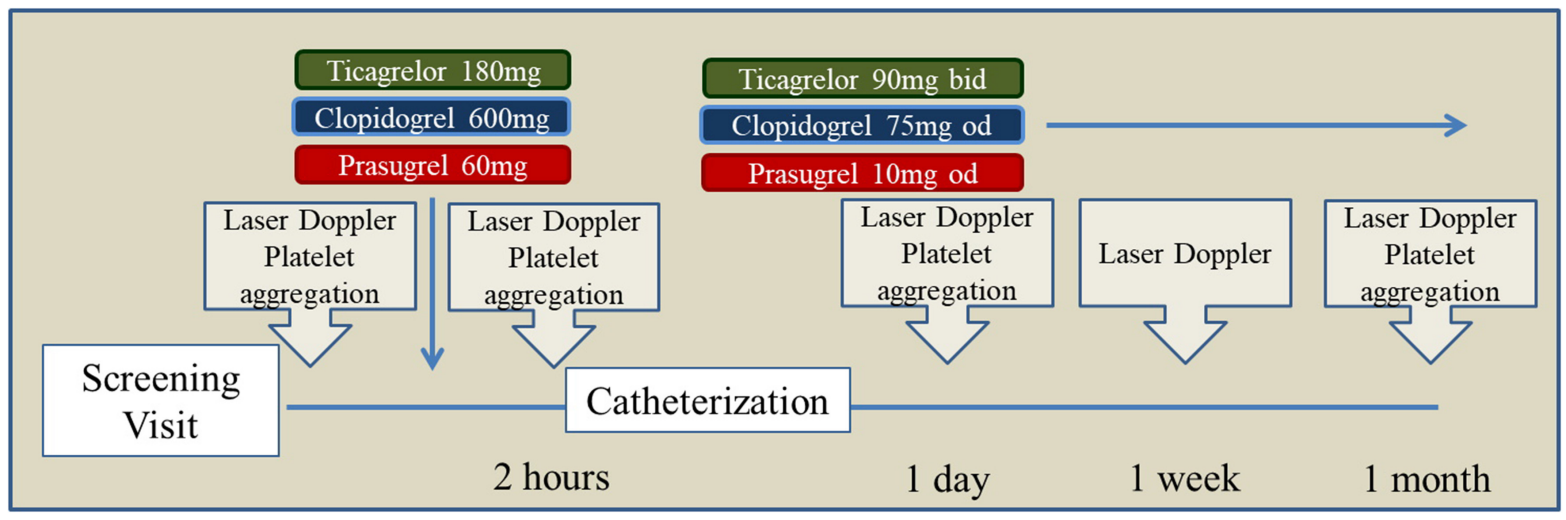
Study protocol.

We report herein on a pre-specified substudy of the EST trial which was only conducted prior to interim analysis of the main trial (10). The schedule of this substudy, as planned in the original submission for funding by the German Ministry, is presented in Figure 1. Its primary endpoint was the change in laser-Doppler post-occlusion reactive hyperemia (PORH%) following the first loading dose of clopidogrel, prasugrel, or ticagrelor and up to 1 month after PCI. Secondary objectives were the changes in microvascular conductance and the existence of a correlation between platelet and microvascular responses to each of the study drug.

### Trial Therapy

For this substudy, study drugs were administered orally as a single loading dose of 600 mg (clopidogrel), 60 mg (prasugrel) or 180 mg (ticagrelor) followed by chronic treatment: clopidogrel in a dosage of 75 mg o.d., 10 mg o.d. prasugrel or 90 mg b.i.d. ticagrelor. Compliance was assessed by pill count in all patients.

### Assessment of Microvascular Function

Subjects were placed in supine position with their arms at heart level. A laser Doppler flowmetry (LDF) probe was placed on the on the palmar side of the metacarpal of the first finger of the right hand using adhesive tape. After 15 min of acclimatization, baseline measurements of microvascular perfusion, blood pressure and heart rate were made. Blood flow was then occluded at the level of the brachial artery using a blood pressure cuff placed around the upper arm inflated to a pressure of 240 mmHg. Ischemia was maintained for 4.5 min. After the blood pressure cuff was released, the PORH response was recorded until the perfusion had decreased to the reference level.

Blood flow was recorded continuously. The amplitude of the PORH was determined as absolute peak and relative change in absolute perfusion units. Data were also expressed as cutaneous vascular conductance, calculated as flux in mV divided by mean arterial pressure in mmHg, a more physiological approach that takes into account differences and variations in blood pressure. The analysis was performed off-line in a random order by staff blinded to the allocation group with signal processing software (PeriSoft 2.5.5; Perimed, Järfälla, Sweden). The reproducibility of the methods has been reported elsewhere (11, 12).

### Platelet Function

Platelet reactivity testing *in vitro* was assessed by ADP-induced aggregation in hirudin-anticoagulated whole blood by using whole blood impedance/multiple electrode aggregometry (Multiplate^®^ analyser and ADPtest, Roche Diagnostics, Rotkreuz Switzerland) (13).

### Statistics

The randomization was performed using SAS. Per protocol, the efficacy analysis was based on a modified intention to treat analysis set, containing all patients who received at least one stent and had at least one PORH measurement during follow up.

Patient characteristics are presented as mean ± standard deviation (SD), median [interquartile range] or number (%) as appropriate. For the primary analysis, since the amplitude of the hyperemic responses did not follow a normal distribution, Kruskal-Wallis test and Mann-Whitney test for between-group comparisons, and Spearman rank correlation test for the relationship between quantitative variables. Two-sided significance tests were used throughout. Details of the statistical analysis of the data collected in this trial were documented in a Statistical Analysis Plan that was generated by the trial statistician and finalized before closing the database and breaking the randomization code. The statistical analysis was conducted by means of Medcalc^®^ (Mariakerke, BE).

### Ethic Committee and Regulatory Approval

The trial was carried out in keeping with legal and regulatory requirements and the protocol was approved by the local ethics committee and by the national authorities. National regulations (Arzneimittelgesetz and the Federal Data Protection Law) were kept. The main EST trial was registered in ClinicalTrials.gov (Identifier: NCT01700322) and EUDRACT-N°.: 2011-005305-73. One-hundred percentage of the data was externally monitored by the Interdisciplinary Center for Clinical Studies at the University Medical Center Mainz, which was also responsible for aspects of patient safety and compliance monitoring, auditing, regulatory.

## Results

### Patient Characteristics

A total of 56 patients [age: 63 [55–67] years, males, 10 diabetics, 27 patients with NSTEMI, 29 with unstable angina] were enrolled in the study. Patient characteristics, including concomitant medications, are described in Tables 1, 2. There were no significant differences among groups.

**Table 1:** Patient characteristics.

	***Treatment group***						
	***Clopidogrel (n = 20)***		***Prasugrel (n = 15)***		***Ticagrelor (n = 21)***		
	***Median***	***IQR***	***Median***	***IQR***	***Median***	***IQR***	***P-value***
Age, years	64	55–71	61	55–65	64	52–65	0.503
Male, *n* (%)	18	(75%)	12	(80%)	17	(81%)	0.129
BMI	27	26–29	29	26–31	29	27–30	0.218
Blood pressure, sytolic, mmHg	130	127–145	133	121–150	136	120–150	0.913
Blood pressure, diastolic, mmHg	80	79–82	80	66–97	80	70–94	0.969
Clinical presentation, NSTEMI (*n*, %)	8	(40%)	7	(47%)	12	(57%)	0.271
Diabetes (*n*, %)	2	(10%)	2	(13%)	6	(29%)	0.119
Hyperlipidemia (*n*, %)	12	(60%)	8	(53%)	12	(57%)	0.857
Hypertension (*n*, %)	13	(65%)	12	(80%)	15	(71%)	0.623
Smoking (*n*, %)	6	(30%)	4	(53%)	10	(48%)	0.156
Former smoker	6	(30%)	7	(47%)	2	(9.5%)	
Family history (*n*, %)	11	(55%)	8	(53%)	12	(57%)	0.974

**Table 2:** Concomitant medications.

***Medications***	***Clopidogrel n = 20***	***Prasugrel*** ***n = 15***	***Ticagrelor*** ***n = 21***	***P***
ASA, *n* (%)	20 (100%)	15 (100%)	21 (100%)	1
ACE-inhibitor, *n* (%)	14 (70%)	9 (60%)	15 (71%)	0.745
ß-Blocker, *n* (%)	16 (80%)	9 (60%)	19 (90%)	0.283
AT-1-Antagonist, *n* (%)	5 (25%)	6 (40%)	7 (33%)	0.130
Ca^2+^-Antagonist, *n* (%)	4 (20%)	4 (27%)	5 (24%)	0.063
Renin inhibitor, *n* (%)	1 (5%)	1 (7%)	0 (0%)	0.151
Statins/ Other anti HLP, *n* (%)	16 (80%)/4 (20%)	14 (93%)/ 1 (7%)	16 (76%)/2 (10%)	0.294
Diuretics, *n* (%)	11 (55%)	7 (47%)	7 (34%)	0.185
PPIs, *n* (%)	10 (50%)	3 (20%)	7 (33%)	0.241
L-Thyroxin, *n* (%)	2 (10%)	1 (7%)	3 (14%)	0.100

### Platelet Aggregatory Reactivity in Response to ADP

Platelet aggregation data are presented in Table 3. There was no difference among groups at screening. ADP-induced platelet aggregation capacity decreased in all groups 2 h after oral loading, with a significant difference among groups (*P* = 0.009; *P* = 0.006 for the comparison prasugrel vs. clopidogrel, *P* = 0.107 for the comparison prasugrel vs. ticagrelor, *P* = 0.048 for the comparison clopidogrel vs. ticagrelor). At one day and 1 month, a difference among groups was maintained (*P* = 0.006 among groups for both timepoint; between group comparisons in Table 3).

**Table 3:** Platelet aggregation, aggregation units.

	***Treatment group***						***P***
	***Clopidogrel (n = 20)***		***Prasugrel (n = 15)***		***Ticagrelor (n = 21)***		
	***Median***	***IQR***	***Median***	***IQR***	***Median***	***IQR***	
*Screening*	51	37–86	60	37–79	61	35–66	
							0.902
*After oral loading*	28	19–60	15	11–21	20	16–27	
							0.009*
*Non-responders (n, %)*	7/20	35%	1/20	5%	0/20	0%	
*1 day after stenting*	13	11–18	8	3–11	11	8–13	
							0.006**
*Non-responders (n, %)*	0/20	0%	0/20	0%	0/20	0%	
*1 Month after stenting*	25	18–34	16	12–19	17	13–22	
							0.006***
*Non-responders (n, %)*	2/20	10%	0/20	0%	0/20	0%	

*Non-responders are defined as Multiplate ADP >46 aggregation units*.

* *: P = 0.006 for the comparison prasugrel vs. clopidogrel, P = 0.107 for the comparison prasugrel vs. ticagrelor, P = 0.048 for the comparison clopidogrel vs. ticagrelor*;

** *: P = 0.002 for the comparison prasugrel vs. clopidogrel, P = 0.051 for the comparison prasugrel vs. ticagrelor, P = 0.136 for the comparison clopidogrel vs. ticagrelor*;

*** *P = 0.009 for the comparison prasugrel vs. clopidogrel, P = 0.657 for the comparison prasugrel vs. ticagrelor, P = 0.009 for the comparison clopidogrel vs. ticagrelor*.

### Microvascular Function

**Figure 2:**
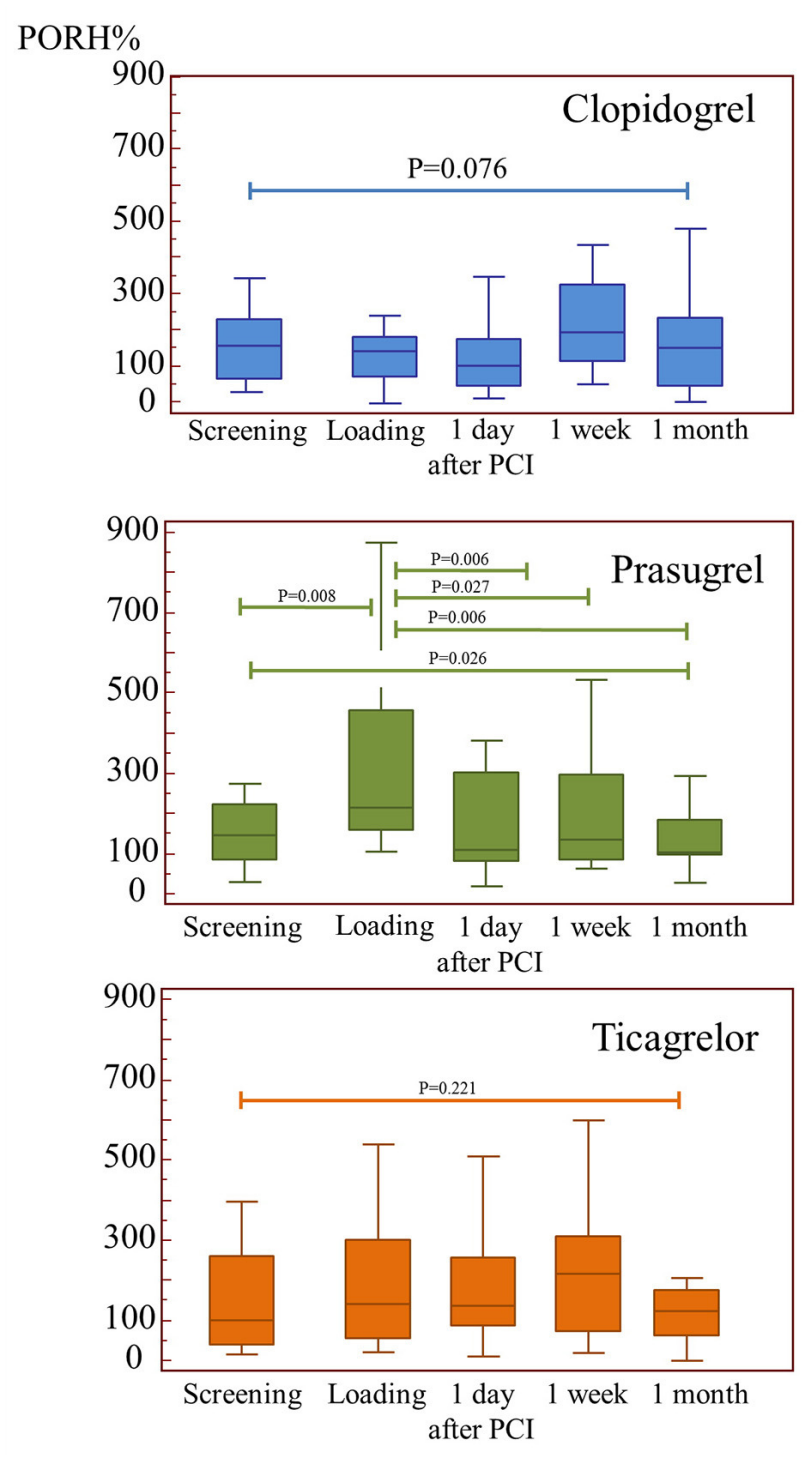
The impact of the three study medications on microvascular function. PORH (post-occlusion reactive hyperemia) was improved acutely after loading by prasugrel, however this effect was lost after percutaneous intervention.

**Figure 3:**
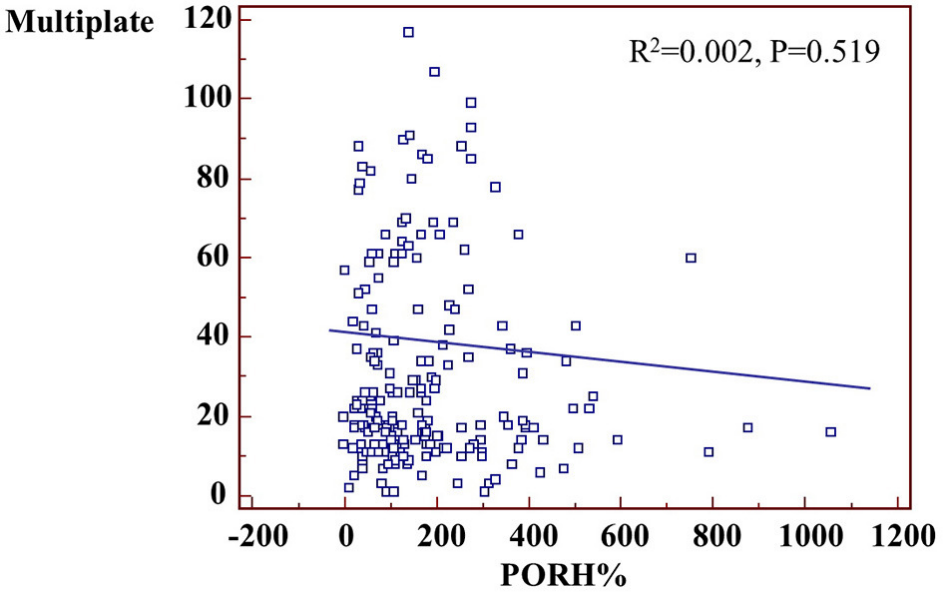
Correlation between platelet activity and PORH.

Treatment effects on microvascular cutaneous perfusion and microvascular conductance are shown in Tables 4, 5 as well as in Figure 2. Resting perfusion data were not different among groups at any time point. After the loading dose of the three medications, PORH% increased in the prasugrel and ticagrelor group [delta changes from screening visit: −24(−53/−5)% for clopidogrel, 79(8/227)% for prasugrel, 54(−33/156)% for ticagrelor, ANOVA *P* = 0.015, *P* = 0.007 for the comparison prasugrel vs. clopidogrel, *P* = 0.329 for the comparison prasugrel vs. ticagrelor and *P* = 0.042 ticagrelor compared to clopidogrel)]. In absolute values, %PORH was highest in the prasugrel compared to clopidogrel group (*P* = 0.003 for the comparison prasugrel vs. clopidogrel), without a difference in the other direct comparisons (*P* = 0.117 for the comparison prasugrel vs. ticagrelor, *P* = 0.415 for the comparison clopidogrel vs. ticagrelor). At one day after PCI, PORH% was decreased in all groups [delta: −30(−89/97)% for clopidogrel, −126(−315/38)% for prasugrel, −40(−158/−104)% *P* = 0.157]. No further differences among the groups were seen during the following visits. In the within-group comparisons, the effect of loading on %PORH was only manifest in the prasugrel group 2 h after loading, with a progressive decrease in the following visits (Figure 2). On two-ways ANOVA, prasugrel (*P* = 0.025) but not ticagrelor (*P* = 0.181) was superior to clopidogrel over the whole study period. Similar changes were observed when microvascular conductance was analyzed (Table 5): reactive hyperemia was significantly larger in the prasugrel group after oral loading and it dropped after coronary stenting in all groups, with a larger change in the prasugrel group. There were no differences among groups during subsequent follow-up (Tables 4, 5). On regression analysis, there was no association between platelet function and microvascular function (*R*^2^ = 0.002, *P* = 0.519, Figure 3). As well, there was no correlation between the delta change in platelet reactivity from screening visit to after oral loading and the delta change in PORH%.

**Table 4:** Microvascular function data.

***Flow (Perfusion Units, in mV)***							***P***
	***Treatment group***						
	***Clopidogrel (n = 20)***		***Prasugrel (n = 15)***		***Ticagrelor (n = 21)***		
	***Median***	***IQR***	***Median***	***IQR***	***Median***	***IQR***	
*Screening*	44	28–74	39	27–68	61	20–135	
*Resting*							0.440
*Peak during Hyperemia*	119	71–173	108	64–146	124	84–197	0.363
*% PORH*	156	65–229	146	86–224	88	40–265	0.502
*After oral loading*	45	29–83	29	19–48	50	19–116	
*Resting*							0.158
*Peak during Hyperemia*	111	75–160	112	64–148	133	73–198	0.572
*% PORH*	140*	70–180	216*	159–457	159*	58–342	0.022**
*1 day after stenting*	59	40–80	31	23–52	40	25–82	
*Resting*							0.091
*Peak during Hyperemia*	138	89–179	102	66–136	92	84–172	0.437
*% PORH*	101	47–177	110†	82–303	139	95–264	0.287
*1 week after stenting*	30	17–60	49	20–75	33	21–75	
*Resting*							0.490
*Peak during Hyperemia*	94	81–135	116	88–192	120	67–173	0.599
*% PORH*	193	113–327	135	86–298	224	90–352	0.726
*1 Month after stenting*	49	27–81	57	30–71	57	26–95	
*Resting*							0.609
*Peak during Hyperemia*	119	96–140	119	91–130	134	9–175	0.436
*% PORH*	152	46–233	106	100–184	125	65–175	0.894

* *: P = 0.015 among groups for the change in %PORH between screening and following oral loading (P = 0.007 for the comparison clopidogrel vs. prasugrel; P = 0.329 for the comparison prasugrel vs. ticagrelor and P = 0.042 for the comparison clopidogrel vs. ticagrelor)*;

** *: P = 0.003 for the comparison prasugrel vs. clopidogrel, P = 0.117 for the comparison prasugrel vs. ticagrelor, P = 0.415 for the comparison clopidogrel vs. ticagrelor*;

† * = 0.026 for the comparison with immediately after loading*.

**Table 5:** Microvascular conductance.

***Vascular conductance (mV/mmHg)***							***P***
	***Treatment group***						
	***Clopidogrel (n = 20)***		***Prasugrel (n = 15)***		***Ticagrelor (n = 21)***		
	***Median***	***IQR***	***Median***	***IQR***	***Median***	***IQR***	
*Screening*	0.449	0.259–0.819	0.360	0.272–0.638	0.750	0.243–1.504	
*Resting*							0.262
*Peak during Hyperemia*	1.263	0.933–1.578	1.095	0.576–1.627	1.373	0.856–2.097	0.210
*% PORH*	154.1	61.4–241.2	138.2	72.5–227.2	89.0	39.7–264.8	0.545
*After oral loading*	0.560	0.345–0.805	0.273	0.190–0.478	0.578	0.239–1.233	
*Resting*							0.05
*Peak during Hyperemia*	1.263	0.933–1.578	1.095	0.576–1.627	1.373	0.856–2.097	0.446
*% PORH*	140.3	73.8–178.4	209.9*	153.7–473.9	158.7	57.9–342.0	0.022
*1 day*	0.616	0.454–0.960	0.275	0.245–0.592	0.418	0.305–0.820	
*Resting*							0.05
*Peak during Hyperemia*	1.261	0.993–1.855	1.061	0.626–1.261	1.088	0.835–1.727	0.200
*% PORH*	97.9‡	43.4–167.2	107.5†	79.4–297.3	138.9‡	95.2–264.9	0.186
*1 week*	0.338	0.190–0.623	0.590	0.354–0.763	0.416	0.230–0.786	
*Resting*							0.438
*Peak during Hyperemia*	1.023	0.750–1.670	1.152	1.020–2.255	1.362	0.882–1.979	0.458
*% PORH*	225.0	107.4–338.9	134.7	94.1–255.5	220.7	78.6–328.6	0.725
*1 Month*	0.493	0.291–0.988	0.530	0.279–0.668	0.633	0.306–0.974	
*Resting*							0.545
*Peak during Hyperemia*	1.307	0.969–1.601	1.250	0.956–1.336	1.290	0.990–1.838	0.320
*% PORH*	148.3	45.0–215.5	106.5	97.8–211.5	124.9	64.6–175.3	0.865

* *P = 0.011 for the post-hoc comparison with clopidogrel, P = 0.122 for the comparison with ticagrelor. P = 0.224 for the comparison ticagrelor and clopidogrel*.

† *P = 0.020 and*

‡ * = 0.5 for the comparison with immediately after loading*.

## Discussion

Platelets have been shown to have a role in both vascular inflammation and atherogenesis (14), and several studies have shown that platelets are an important source of oxidative stress in acute coronary syndromes and in the setting of PCI (15, 16). In patients with stable angina or shortly after an ACS, antiplatelet agents improve vascular endothelial function (2, 3, 17), an effect that is however lost upon prolonged treatment with clopidogrel (4). In the present study, we compare the effect of three antiplatelet agents of different potency on markers of microvascular function in patients undergoing PCI for ACS. We find that acutely (before PCI), antiplatelet therapy with prasugrel but not clopidogrel improves microvascular function, while ticagrelor appears to have an intermediate effect. Although prasugrel was also a more potent inhibitor of platelet function, there was no statistical association between platelet inhibition and improvement of microvascular reactivity, potentially suggesting a pleiotropic effect of antiplatelet agents. Importantly, this effect on cutaneous microvascular function was lost one day after PCI and during subsequent follow-up. Of note, the current data do not allow any conclusion with respect to the long-term benefits of P2Y12 inhibitors on microvascular damage.

Cutaneous microvascular function provides an easily accessible surrogate for the myocardial circulation. Laser Doppler measures reflect the status of microcirculation in other vascular beds (18, 19), are impaired in the setting of cardiovascular disease (18), and correlate with left ventricular strain (20). These techniques have been used to predict vascular dysfunction or to evaluate the effect of drugs on microcirculation in various diseases (21–26). In particular, post-occlusion reactive hyperemia is a complex phenomenon that is determined by a number of mediators, including axon reflex response, local mediators such as the endothelium-derived hyperpolarizing factor, prostaglandins and nitric oxide (27).

Several studies have reported an association between antiplatelet therapy and improvements in (micro)vascular function. Of note, this pleiotropic effect of P2Y_12_ inhibitors appears to be dependent on the drug and dose administered and on the timing of the administration. In the Armyda trial, 150 mg clopidogrel improved peripheral artery endothelial function and reduced C-reactive protein as compared to standard 75 mg (28). In the paper by Rudolph et al. (29), prasugrel, but not clopidogrel, improved conduit artery endothelial function and reduced sCD40 ligand and RANTES levels while increasing nitrite levels three months after PCI for unstable angina. In the EST trial, all three P2Y_12_ inhibitors acutely improved radial artery flow-mediated dilation prasugrel, but this effect was lost after stenting in patients receiving clopidogrel and ticagrelor. In contrast, in the paper by Jeong et al. ticagrelor showed to be superior as compared to prasugrel in improving conduit artery endothelial function and markers of vascular inflammation (17). In the HI-TECH study, none of the three antiplatelet agents improved reactive hyperemia at 1–2 h or 30 days after administration (30). Finally, previous studies showed that coronary stenting is associated with increased platelet aggregation, leukocyte activation, increased oxidative stress, and red blood cell aggregation (1, 31). In the current study, we investigated the effect of antiplatelet agents and stenting in the setting of unstable angina or NSTEMI, which might explain some of the differences with previous literature.

In sum, we observed an acute improvement in microvascular function in patients undergoing stenting for an acute coronary syndrome 2 h after administration of prasugrel and, to a lesser extent, ticagrelor. Of note, this effect was lost at one and 30 days, i.e., immediately and during short-term follow-up after stent implantation.

## Limitations

This randomized, blinded study relies on a small sample size and needs further replication. The trial was based on a parallel design without cross-over, which was the only possible approach in an acute setting. Second, a placebo control was impossible as it would have exposed the patients to an unacceptable risk of acute stent thrombosis. Whether stenting would have reduced PORH% in this hypothetical placebo group could therefore not be investigated. Finally, all patients received acetyl salicylic acid (as per clinical guidelines) before coronary reperfusion. Acetyl salicylic acid inhibits prostaglandin synthesis, thus affecting (micro)vascular reactivity and platelet activity. While low-dose aspirin may impair acetylcholine and thermal responses measured by laser Doppler, hyperemic responses to ischemia do not seem to be affected (32). Finally, platelet function was assessed with multiplate. Wheter other methods would have provided different outcomes remains unknown.

## Conclusions

Various randomized clinical trials have shown a significant benefit of potent P2Y_12_ inhibitors over clopidogrel in ACS and stable high-risk patients. The current data show that, when administered pre-PCI, prasugrel and (less consistently) ticagrelor improve microvascular function, an effect that is however lost upon short-term follow-up.

## Clinical Perspectives

Percutaneous coronary interventions cause vascular damage, inflammation and endothelial dysfunction, a phenomenon that is (also) mediated by activation of platelets. Potent P2Y_12_ inhibitors reduce the incidence of cardiovascular events in patients with acute coronary syndromes, and we recently showed that prasugrel, as compared to ticagrelor, also prevents PCI-induced endothelial dysfunction. The current data show that, when administered before PCI, prasugrel and (to a lesser extent) ticagrelor improve microvascular function, with a larger effect of the more potent prasugrel.

## Data Availability Statement

The original contributions presented in the study are included in the article/Supplementary Material, further inquiries can be directed to the corresponding author/s.

## Ethics Statement

The studies involving human participants were reviewed and approved by Landesärztekammer Mainz. The patients/participants provided their written informed consent to participate in this study.

## Author Contributions

BS, KJ, CW, and TG participated in data acquisition, analysis, writing and revision of the manuscript. TM and KL revised the manuscript. All authors contributed to the article and approved the submitted version.

## Funding

The research was funded by the German Ministry for Research through the IFB project center for thrombosis and hemostasis at the University Medical Center Mainz.

## Conflict of Interest

TG has participated in symposia and meetings sponsored by Daiichi-Sankyo and by Astra Zeneca. The remaining authors declare that the research was conducted in the absence of any commercial or financial relationships that could be construed as a potential conflict of interest.

## Publisher's Note

All claims expressed in this article are solely those of the authors and do not necessarily represent those of their affiliated organizations, or those of the publisher, the editors and the reviewers. Any product that may be evaluated in this article, or claim that may be made by its manufacturer, is not guaranteed or endorsed by the publisher.

## Supplementary Material

Supplementary Figure 1 The Laser-Doppler method. More information on the method can be found in [33–36].

Supplementary Figure 2 Correlation between platelet activity and PORH 2 h after loading dose.
