# Supplementary figures and images for: Effects of Clopidogrel, Prasugrel and Ticagrelor on Microvascular Function and Platelet Reactivity in Patients With Acute Coronary Syndrome Undergoing Coronary Artery Stenting. A Randomized, Blinded, Parallel Group Trial
Source: Front Cardiovasc Med. 2021 Dec 13;8:780605. doi: 10.3389/fcvm.2021.780605 (PMC8710519; doi:10.3389/fcvm.2021.780605)

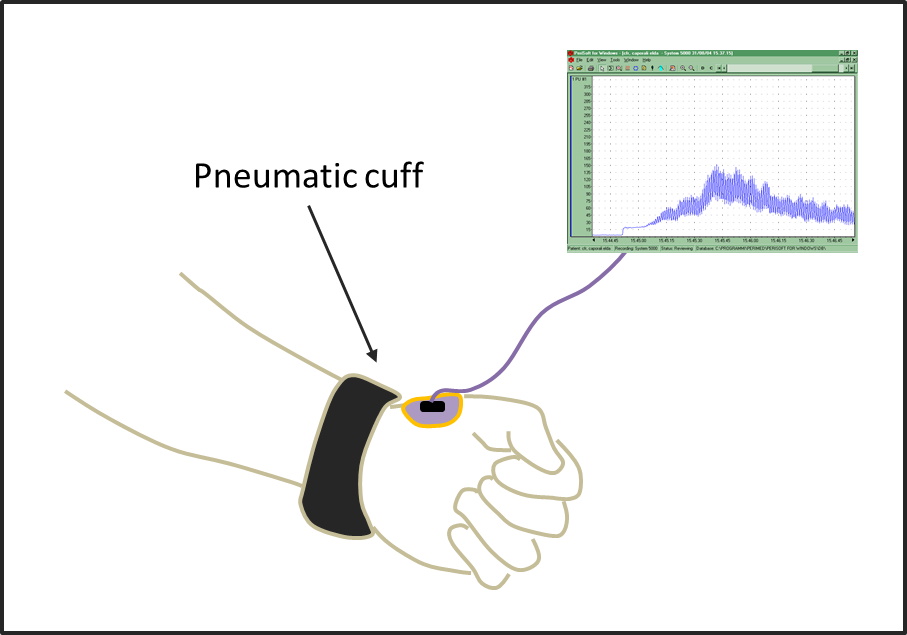

Supplement: Supplementary Figure 1 — The Laser-Doppler method. More information on the method can be found in [33–36]. [file Image_1.JPEG]

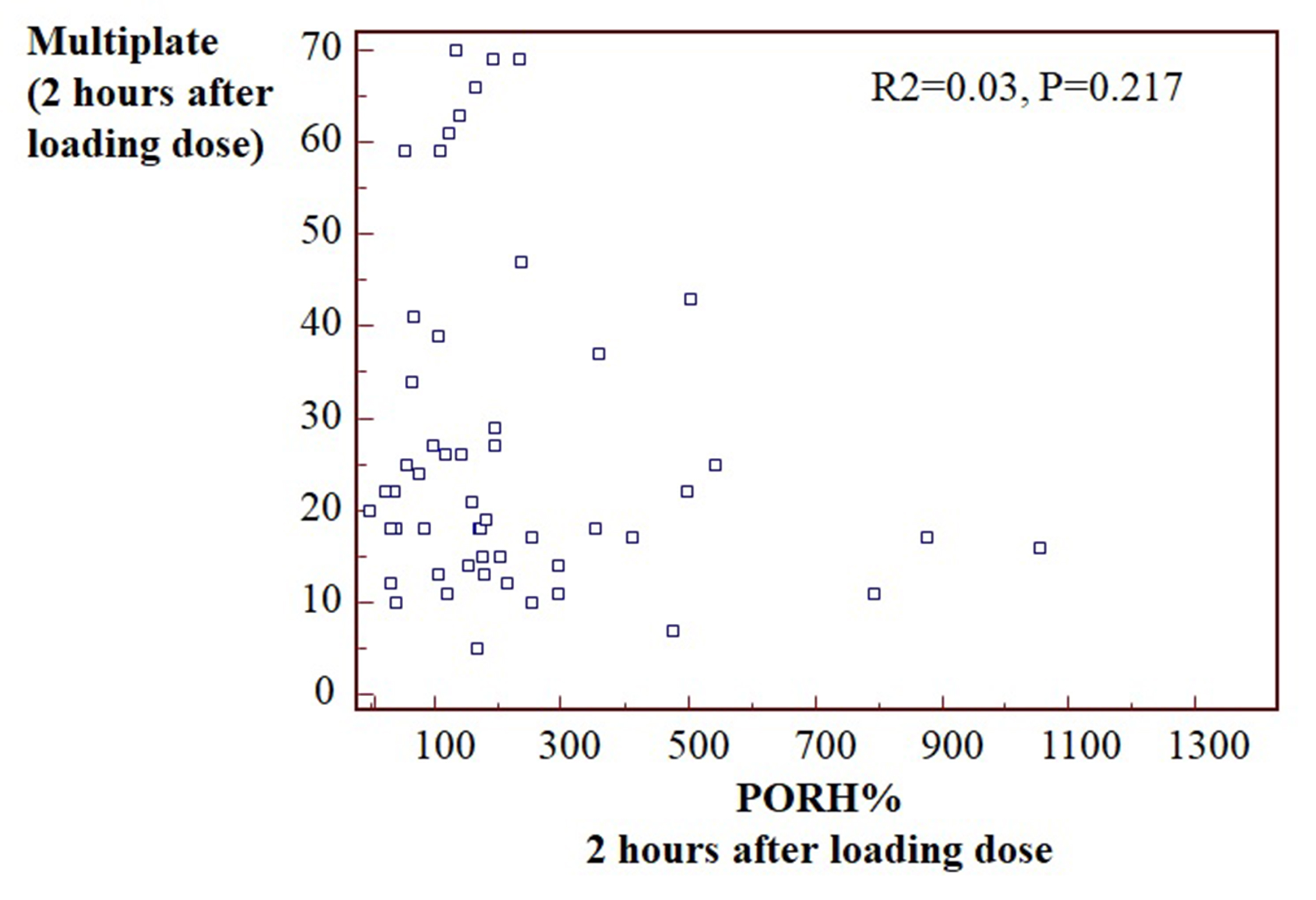

Supplement: Supplementary Figure 2 — Correlation between platelet activity and PORH 2 h after loading dose. [file Image_2.JPEG]
